# BTK Inhibitors Impair Platelet-Mediated Antifungal Activity

**DOI:** 10.3390/cells11061003

**Published:** 2022-03-16

**Authors:** Vincenzo Nasillo, Ivana Lagreca, Daniela Vallerini, Patrizia Barozzi, Giovanni Riva, Monica Maccaferri, Ambra Paolini, Fabio Forghieri, Stefania Fiorcari, Rossana Maffei, Silvia Martinelli, Claudio Giacinto Atene, Ilaria Castelli, Roberto Marasca, Leonardo Potenza, Patrizia Comoli, Rossella Manfredini, Enrico Tagliafico, Tommaso Trenti, Mario Luppi

**Affiliations:** 1 Diagnostic Hematology and Clinical Genomics, Department of Laboratory Medicine and Pathology, AUSL/AOU Modena, 41124 Modena, Italy; g.riva@ausl.mo.it (G.R.); enrico.tagliafico@unimore.it (E.T.); t.trenti@ausl.mo.it (T.T.); 2 Section of Hematology, Department of Medical and Surgical Sciences, University of Modena and Reggio Emilia, AOU Modena, 41124 Modena, Italy; ivana.lagreca@unimore.it (I.L.); daniela.vallerini@unimore.it (D.V.); patrizia.barozzi@unimore.it (P.B.); maccaferri.monica@aou.mo.it (M.M.); paolini.ambra@aou.mo.it (A.P.); fabio.forghieri@unimore.it (F.F.); stefania.fiorcari@unimore.it (S.F.); rossana.maffei@unimore.it (R.M.); silvia.martinelli@unimore.it (S.M.); claatene@unimore.it (C.G.A.); ilaria.castelli@unimore.it (I.C.); roberto.marasca@unimore.it (R.M.); leonardo.potenza@unimore.it (L.P.); mario.luppi@unimore.it (M.L.); 3 Pediatric Hematology/Oncology Unit and Cell Factory, Istituto di Ricovero e Cura a Carattere Scientifico (IRCCS) Policlinico San Matteo, 27100 Pavia, Italy; pcomoli@smatteo.pv.it; 4 Centre for Regenerative Medicine “S. Ferrari”, University of Modena and Reggio Emilia, 41125 Modena, Italy; rossella.manfredini@unimore.it

**Keywords:** BTK inhibitors, platelets, ibrutinib, acalabrutinib, CLL, invasive fungal infections, molds, *Aspergillus*

## Abstract

In recent years, the introduction of new drugs targeting Bruton’s tyrosine kinase (BTK) has allowed dramatic improvement in the prognosis of patients with chronic lymphocytic leukemia (CLL) and other B-cell neoplasms. Although these small molecules were initially considered less immunosuppressive than chemoimmunotherapy, an increasing number of reports have described the occurrence of unexpected opportunistic fungal infections, in particular invasive aspergillosis (IA). BTK represents a crucial molecule in several signaling pathways depending on different immune receptors. Based on a variety of specific off-target effects on innate immunity, namely on neutrophils, monocytes, pulmonary macrophages, and nurse-like cells, ibrutinib has been proposed as a new host factor for the definition of probable invasive pulmonary mold disease. The role of platelets in the control of fungal growth, through granule-dependent mechanisms, was described in vitro almost two decades ago and is, so far, neglected by experts in the field of clinical management of IA. In the present study, we confirm the antifungal role of platelets, and we show, for the first time, that the exposure to BTK inhibitors impairs several immune functions of platelets in response to *Aspergillus fumigatus*, i.e., the ability to adhere to conidia, activation (as indicated by reduced expression of P-selectin), and direct killing activity. In conclusion, our experimental data suggest that antiplatelet effects of BTK inhibitors may contribute to an increased risk for IA in CLL patients.

## 1. Introduction

In recent years, new compounds targeting Bruton’s tyrosine kinase (BTK) have successfully been introduced in the treatment algorithm of chronic lymphocytic leukemia (CLL) and other B-cell neoplasms, pioneering a major therapeutic shift from chemoimmunotherapy to targeted therapy [1]. Although BTK inhibitors (BTKis) were initially considered less immunosuppressive than chemoimmunotherapy, an increasing number of reports have described the occurrence of infectious complications in ibrutinib-treated patients [2,3,4,5,6], with an unexpectedly high incidence of opportunistic invasive fungal infections (IFIs), mainly invasive aspergillosis (IA) [7,8,9,10,11,12,13,14,15]. Peculiarly, cases of IA occurring under ibrutinib are characterized by an early onset (usually within the first 6 months of treatment) and high rate of central nervous system (CNS) involvement [16,17]. Several lines of evidence suggest that the increased risk for IFIs cannot strictly be attributed to the effects of ibrutinib on the humoral immunity nor to the BTK inhibition alone, raising the possibility of off-target effects as major contributors [17,18,19].

Ibrutinib is an irreversible BTKi affecting pathways downstream of B-cell receptor (BCR) in malignant B lymphocytes, but has also potent effects on the normal cells of the immune system, thus exerting immunomodulatory activity [20]. BTK represents a crucial molecule in the transmission of signaling cascades from different innate immune receptors, including Toll-like receptors (TLRs), Fc-gamma receptors (FcγRs), triggering receptor expressed on myeloid cells-1 (TREM-1), Dectin-1, and CD11b/CD18 (complement receptor 3, CR3), which allow the recognition of fungi by the innate arm of the immune system [21]. Neutrophils and macrophages, which coexpress BTK and TEC (both targeted by ibrutinib), represent the first line of defense against human fungal infections, acting chiefly through phagocytosis and direct pathogen killing [22,23].

The role of neutrophils is essential for *Aspergillus fumigatus* (*A. fumigatus*) clearance, especially in the early phases of fungal infection, and neutropenia constitutes the principal risk factor for IA [24,25]. BTK is required for E-selectin-mediated slow rolling of neutrophils on inflamed endothelial cells [26,27], as well as for signal transmission from pattern recognition receptors (PRRs) detecting pathogen-associated molecular patterns (PAMPs), i.e., β-glucans, mannans, and chitins [28,29,30]. Once at the site of fungal infection, neutrophils release granule proteins, radical oxygen species (ROS), and neutrophil extracellular traps (NETs) [25,31]. In mouse models, BTK-deficient neutrophils show defective expression of granule proteins and impaired release of ROS and nitric oxide in response to different inflammatory stimuli [32,33]. In line with this, a recent study by Blez and colleagues clearly demonstrated that ibrutinib blunts anti-*A. fumigatus* responses of neutrophils both in vitro and in vivo [34].

Macrophages can engulf and degrade fungal conidia and hyphae, after recognition of specific PAMPs by different PRRs. In addition, macrophages can recruit neutrophils by releasing cytokines, such as tumor necrosis factor α (TNF-α) and interleukin 1β (IL-1β) [22,23]. Several studies have displayed the involvement of BTK in FcγR-mediated phagocytosis in macrophages. The activation of TLR9 by phagocytosis of *A. fumigatus* conidia drives the BTK-calcineurin–NFAT signaling pathway, which is crucial for TNF-α production by macrophages and subsequent chemotaxis of neutrophils to the airways during pulmonary aspergillosis [35]. Consistently, BTK-deficient macrophages exhibit impaired fungal phagocytosis, disruption of intracellular signal propagation after PAMPs-PRRs binding, and reduced cytokine release [32]. Ibrutinib targets BTK expressed in CLL-associated macrophages (also known as nurse-like cells, NLCs), exacerbating their immunosuppressive profile through polarization toward M2-type macrophages, showing impaired phagocytic activity [36,37]. Moreover, ibrutinib was found to inhibit NFAT and NF-κB responses in monocyte-derived human macrophages, thus hampering the ability to counteract the fungal growth, albeit without affecting the phagocytic activity [38]. Similarly, a reduction in FcγR-mediated cytokine production, but not phagocytic ability, was observed in circulating monocytes treated in vitro with ibrutinib [39]. By extending these results, our group demonstrated that the exposure to either ibrutinib or acalabrutinib determines an exacerbation of an immunosuppressive profile, a reduction in phagocytosis, and a significant drop in the secretion of inflammatory cytokines, both in NLCs and circulating monocytes from CLL patients and healthy donors, leading to a failure in counteracting conidia germination [40].

Besides neutrophils and monocyte/macrophage populations, platelets (coexpressing BTK and TEC) represent a crucial cell type involved in the host’s innate immune responses. Platelets are equipped with several innate immune receptors, including TLRs and FcγRIIA, which bestow the ability to recognize pathogens [41]. Other molecules, such as P-selectin (CD62P), glycoprotein IV (GPIV), GPIb (CD42b), CD40L, and integrin αIIbβ3, enable the interactions between platelets and leukocytes [42,43]. This process leads neutrophils to produce ROS and form NETs [44]. Furthermore, platelets express soluble molecules, comprising chemokines (e.g., CCL3, CXCL4, CCL5, and CXCL7) and cytokines (e.g., IL-1β and TGF-β) [45]. These factors are present in platelet granules but can also be synthesized de novo. Such a broad armamentarium of immune receptors, adhesion molecules, and soluble factors makes platelets full-fledged innate immune cells that can drive inflammation and mediate pathogen clearance by different mechanisms [45,46,47,48]. Several studies have investigated the role of platelets in the control of fungal infections. The expression of CD62P and other molecules indicative of platelet activation (i.e., CD63, RANTES, or CD40L) was found to be increased following platelet exposure to hyphae or conidia [49,50]. Interestingly, Perkhofer et al. demonstrated that human platelets attenuate *Aspergillus* species in vitro through granule-dependent mechanisms [51]. The same group also showed that the combination of platelets and anidulafungin significantly reduces the germination rate of *A. fumigatus*, alters the hyphal elongation, and downregulates the *fks* gene, encoding for β-D-glucan, an essential component of the cell wall [52]. In addition, *A. fumigatus* antigens seem to affect platelet aggregation in vitro through the deposition of complement factors [53]. Further data suggest that platelets could trigger coagulopathy and activate neutrophils during *A. fumigatus* infection [54,55,56]. In line with these findings, thrombocytopenia constitutes a risk factor for fungal infections in liver transplant recipients [57] and a predictor of outcome in neutropenia-related IA [58]. Since BTKis are known to hinder platelets’ functions through the inhibition of both BTK and TEC [59,60], we hypothesized that defective thrombocyte-mediated immune responses may contribute to the increased risk of IFIs in treated patients. In order to characterize the specific effects of the pharmacological inhibition of BTK on antimold innate immune responses mediated by platelets, we performed a broad functional in vitro and in vivo analysis of samples from both CLL patients and healthy donors.

## 2. Materials and Methods

### 2.1. Patients and Samples

**Figure 1:**
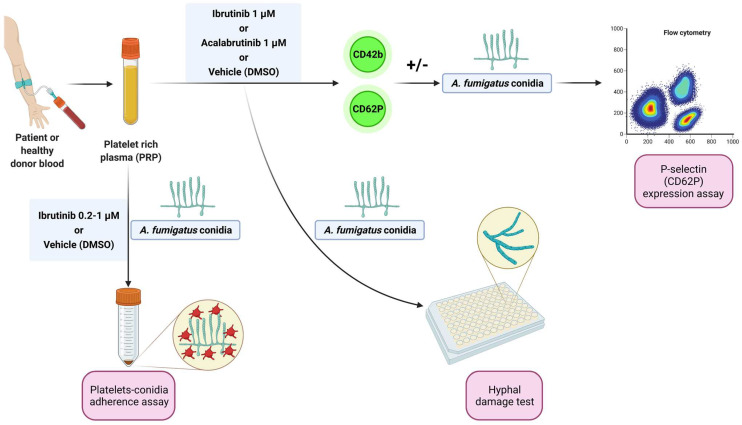
Experimental workflow. Platelet-rich plasma (PRP) from healthy donors and treatment-naïve CLL patients was treated with ibrutinib or acalabrutinib or vehicle (DMSO) for 60 min at 37 °C. Platelet adhesion to conidia was assessed by a spectrophotometric method. Surface expression of CD62P (P-selectin) was evaluated by flow cytometry at baseline and following stimulation by *A. fumigatus* conidia. Platelet-induced hyphal damage was measured by performing a colorimetric assay with XTT. Platelet-mediated antifungal activity was also evaluated in CLL patients before and after ibrutinib administration by cytofluorimetric analysis of P-selectin expression in response to *A. fumigatus* conidia.

Blood samples from patients matching standard diagnostic criteria for CLL were obtained from the Hematology Unit of Modena Hospital, Italy. Written informed consent was obtained in accordance with the Declaration of Helsinki, and the study was approved by the local Institutional Review Board (protocol 63/11 CE AVEN). Peripheral blood samples from healthy donors, treatment-naïve CLL patients, and CLL patients under ibrutinib were centrifuged at 900 rpm for 15 min to obtain platelet-rich plasma (PRP). Before the evaluations, PRP was treated with ibrutinib or acalabrutinib (1 µM) or vehicle (DMSO) for 1 h at 37 °C. For some experiments, increasing concentrations (0.2 µM, 0.5 µM, and 1 µM) of ibrutinib were used. The experimental workflow is summarized in Figure 1.

### 2.2. Platelets–Conidia Adherence Assay

The adherence of platelets to *A. fumigatus* conidia was determined by a previously described spectrophotometric method [49]. In short, ibrutinib-treated or untreated platelets were incubated with *A. fumigatus* conidia at a platelets-to-conidia (effector-to-target, E:T) ratio of 100:1 for 30 min at 37 °C and centrifuged at 500 rpm for 5 min at 4 °C. Platelets alone and conidia alone were used as controls. The 700 nm optical density (OD_700_) of supernatant was determined spectrophotometrically, and the percentage of platelet adherence was calculated by the following formula: {1 − OD_700_ reaction supernatant/[0.5 × (OD_700_ conidia supernatant + OD_700_ platelet supernatant)]} × 100.

### 2.3. P-Selectin Expression Assay

PRP treated with ibrutinib, acalabrutinib, or vehicle was labeled with anti-CD42b and anti-CD62P antibody. CD42b is a membrane glycoprotein constitutively expressed on platelet surface and is useful in identifying platelet population, while CD62P is a marker of activated platelets. Labeled platelets were then incubated with swollen, heat-inactivated *A. fumigatus* conidia at an E:T ratio of 100:1. After 30, 60, 90, 150, 210, and 270 min, samples were acquired and analyzed on a BD Accury C6 flow cytometry. Analysis was performed gating CD42b+ cells and analyzing the mean fluorescence intensity in the positive CD62P population. Results are reported as percentages of CD62b expression normalized on DMSO-treated unstimulated platelets (100%). Normalization was performed by dividing the value of ibrutinib- or acalabrutinib-treated, stimulated or unstimulated samples to the value of the corresponding DMSO-treated unstimulated samples and multiplying by 100.

### 2.4. XTT Assay

*A. fumigatus* conidia were incubated in 96-well plates for 16 h to produce hyphae, with ibrutinib-treated, acalabrutinib-treated, or vehicle-treated platelets, at an E:T ratio of 100:1. Conidia alone were used as positive control. Each experimental condition was performed in triplicate. Then, platelets were lysed hypotonically with H_2_O and hyphae were incubated for 1 h at 37 °C with 2,3-bis[2-methoxy-4-nitro-5-sulfophenyl]2H-tetrazolium-5-carboxyanilide sodium salt (XTT; Sigma, St. Louis, MO, USA) plus coenzyme Q0 (2,3-dimethoxy-5-methyl-1,4-benzoquinone; Sigma) for colorimetric measurement of hyphal metabolic activity. Absorbance was determined at 450 nm using an enzyme-linked immunosorbent assay plate reader, and antifungal activity was calculated as the percentage of hyphal damage according to the equation: [1 − (X/C)] × 100, where X is optical density of test wells and C is optical density of control wells with hyphae only.

### 2.5. Statistical Analyses

Data were analyzed using SPSS version 20.0 (SPSS, Chicago, IL, USA). *p* values were calculated by Student *t* test (* *p* < 0.05; ** *p* < 0.01; *** *p* < 0.001; **** *p* < 0.0001). Data are presented as mean, and standard error of the mean (SEM) is depicted as error bars.

## 3. Results

### 3.1. Ibrutinib Inhibits the Ability of Platelets to Adhere to Conidia

**Figure 2:**
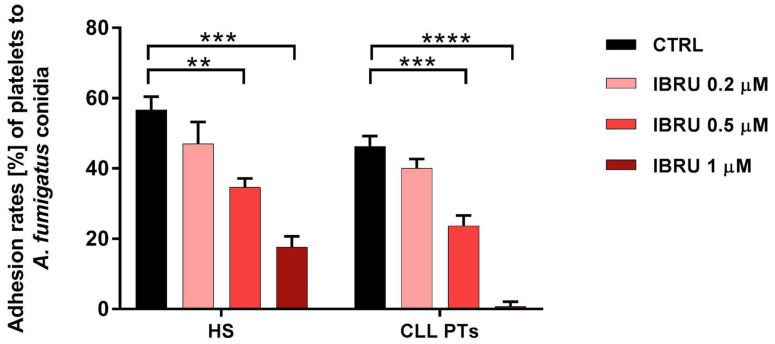
Ibrutinib inhibits adhesion of platelets to conidia. Platelets, treated or not treated with three different concentrations of ibrutinib (0.2 μM, 0.5 μM, and 1 μM), were incubated with *A. fumigatus* conidia, at an effector-to-target (E:T) ratio of 100:1 for 30 min at 37 °C, and then centrifuged at low intensity. Then, the OD_700_ of the supernatant was determined by spectrophotometer, and the percentage of platelet adhesion was calculated in relation to the OD_700_ of platelets and conidia alone (** *p* < 0.01; *** *p* < 0.001; **** *p* < 0.0001). HS, healthy subjects; PTs, patients; CTRL, control; IBRU, ibrutinib.

The ability of platelets to adhere to conidia was assessed in six healthy subjects and six CLL patients by spectrophotometric methods. Our data confirmed the ability of platelets to adhere to conidia in both healthy subjects and CLL patients, with mean adhesion rates of 56.6% (±3.7%) and 46% (±3%), respectively. Treatment with ibrutinib resulted in a significant reduction in adhesion ability in a dose-dependent manner. Specifically, in healthy subjects, mean values of 47% (±6.2%), 34.6% (±2.5%), and 17% (±3%) were found in the presence of ibrutinib at 0.2 μM, 0.5 μM, and 1 μM, respectively. In patients with CLL, the adhesion rate decreased to 40% (±2.5%), 23% (±2.9%), and 1% (±1.3%), in the presence of ibrutinib at 0.2 μM, 0.5 μM, and 1 μM, respectively (Figure 2).

### 3.2. Ibrutinib and Acalabrutinib Reduce P-Selectin Expression on Platelets in Response to A. fumigatus Conidia

**Figure 3:**
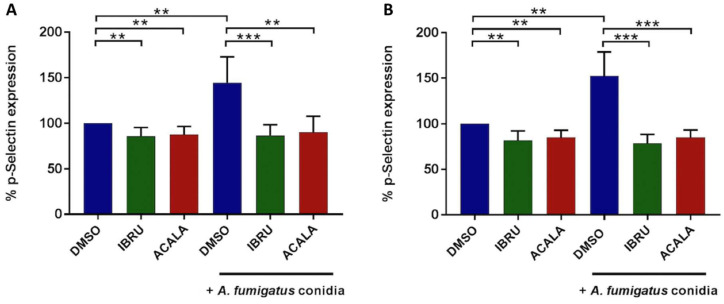
In vitro effects of ibrutinib and acalabrutinib on platelets’ degranulation in response to *A. fumigatus* conidia. Platelet-rich plasma (PRP) from healthy volunteers (**A**) and treatment-naïve CLL patients (**B**) was treated with 1 μM ibrutinib (IBRU), acalabrutinib (ACALA), or vehicle (DMSO) for 1 h at 37 °C and stimulated or not stimulated with *A. fumigatus* conidia at an effector-to-target (E:T) ratio of 100:1 for 30, 60, 90, 120, 180, or 270 min. Platelet activation was detected by flow cytometry, labeling samples with PE-conjugated anti-CD42b antibody, a surface marker constitutively expressed on platelets, and with an FITC-conjugated anti-CD62P/P-selectin antibody, a marker of alpha granule secretion. Results are reported as percentages of CD62b expression normalized on DMSO-treated unstimulated platelets (** *p* < 0.01; *** *p* < 0.001).

To assess whether BTK inhibition affects *A. fumigatus*-induced platelet activation, surface expression of CD62P (P-selectin), a marker of alpha granule secretion, was evaluated by flow cytometry at baseline and following stimulation with *A. fumigatus* conidia. We first performed in vitro experiments on platelet-rich plasma (PRP) from six healthy donors and six treatment-naïve CLL patients. Platelet interaction with conidia strongly induced P-selectin expression on platelet surface, both in healthy volunteers (mean CD62P expression 144%, range 107–184%) and CLL patients (mean 153%, range 126–185%), with different exposure times according to individual patient variability, ranging from 90 to 270 min. P-selectin exposure induced by conidia was significantly reduced in the presence of either ibrutinib or acalabrutinib. In detail, in ibrutinib-treated platelets, mean CD62P expression was 86% (range 65–102%) and 79% (range 70–94%), in healthy donors and CLL patients, respectively. Similarly, in acalabrutinib-treated platelets, mean CD62P expression was 91% (range 67–115%) and 85% (range 75–94%) in healthy donors and CLL patients, respectively. Basal CD62P expression was also significantly reduced in ibrutinib- and acalabrutinib-treated unstimulated platelets compared to controls in both groups (Figure 3). In contrast, response to 10 μM of thrombin receptor agonist peptide (TRAP) was not altered (data not shown).

### 3.3. Ibrutinib and Acalabrutinib Hamper Platelet-Mediated Hyphal Damage

**Figure 4:**
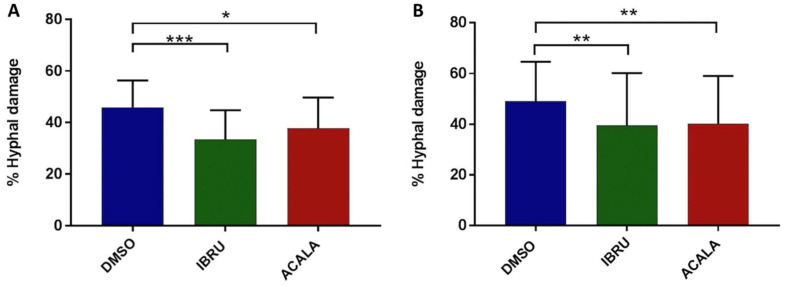
In vitro effects of ibrutinib and acalabrutinib on platelet-mediated hyphal damage. Platelet-rich plasma (PRP) from healthy volunteers (**A**) and treatment-naïve CLL patients (**B**) was treated with 1 μM ibrutinib (IBRU), acalabrutinib (ACALA), or vehicle (DMSO) for 1 h at 37 °C. *A. fumigatus* conidia were incubated for 16 h at 37 °C in RPMI medium plus 1% sodium pyruvate to produce hyphae with or without platelets, at an effector-to-target (E:T) ratio of 100:1. For measurement of hyphal metabolic activity, XTT salt plus 40 μg/mL coenzyme Q was added. Absorbance was determined at 450 nm using an enzyme-linked immunosorbent assay plate reader, and antifungal activity was calculated as the percentage of hyphal damage equal to [1 − (X/C)] × 100, where X is the optical density of test well and C is the optical density of control wells with hyphae only (* *p* < 0.05; ** *p* < 0.01; *** *p* < 0.001).

To further explore the effects of BTK inhibition on antifungal activity, we measured hyphal damage induced by ibrutinib, acalabrutinib, or vehicle-treated platelets by performing a colorimetric assay with XTT. Firstly, we confirmed the ability of platelets to impair germination of conidia and hyphal elongation by counteracting the fungal metabolic activity. Indeed, vehicle-treated platelets from six healthy donors and six CLL patients induced 46% (range 28–58%) and 44% (range 30–58%) mean hyphal damage, respectively. Moreover, we found that ibrutinib- and acalabrutinib-treated platelets showed a statistically significant reduced capacity to induce hyphal damage compared with vehicle-treated platelets. In particular, ibrutinib-treated platelets induced 34% (range 17–50%) and 33% (range 12–51%) mean hyphal damage in healthy volunteers and CLL patients, respectively. Similarly, acalabrutinib-treated platelets induced 38% (range 24–55%) and 34% (range 17–51%) mean hyphal damage in healthy donors and CLL patients, respectively (Figure 4).

### 3.4. Platelet-Mediated Antifungal Activity Decreases during Ibrutinib Treatment in CLL Patients

**Figure 5:**
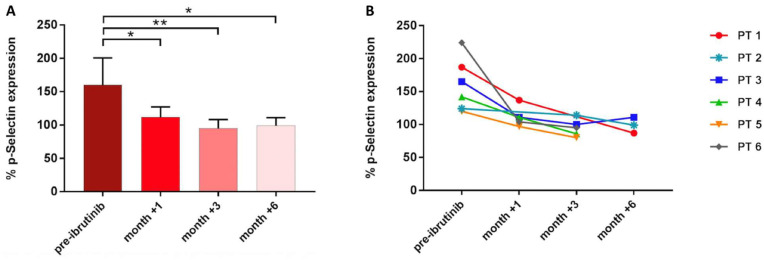
Platelet-mediated antifungal activity in CLL patients (PT) under ibrutinib. Bar (**A**) and line graph (**B**) showing P-selectin expression in samples collected before starting ibrutinib and during the course of treatment (* *p* < 0.05; ** *p* < 0.01).

Next, to verify the clinical relevance of these findings, we evaluated platelet-mediated antifungal activity in six CLL patients before receiving ibrutinib and after 1, 3, and 6 months during the course of treatment. Before treatment, P-selectin expression on the platelet surface was induced in response to *A. fumigatus* conidia (mean CD62P expression 160%, range 120–224%). In contrast, after 1, 3, and 6 months of therapy, *A. fumigatus*-induced P-selectin mean expression was significantly reduced: 124% (range 97–137%) at month +1, 95% (range 80–114%) at month +3, and 99% (range 87–111%) at month +6 (Figure 5).

## 4. Discussion

The lesson learned from IFIs, unexpectedly arising in patients treated with ibrutinib, the first-in-class BTKi, made physicians aware of the potential clinical consequences deriving from the pharmacologic inhibition of such a pleiotropic kinase, as well as from the off-target effects related to this (not so specifically) targeted drug. Indeed, BTK, widely acknowledged as a pivotal B-cell regulator, is expressed in nearly all immune cells, where it is essentially involved in several signaling pathways downstream of different immune receptors, thus mediating different innate immune functions, such as (i) PRR-dependent recognition of infectious agents; (ii) maturation, recruitment, and activation of neutrophils, monocytes, macrophages, and platelets; and (iii) NLRP3 inflammasome activation [21].

Several studies have demonstrated that BTK inhibition by either ibrutinib or acalabrutinib can compromise the ability of neutrophils, monocytes, normal human macrophages, and NLCs to counteract the fungal growth in vitro [34,38,40,61]. However, acalabrutinib has not yet been associated with an increased risk for IFIs in treated patients and has already been shown to have different influences on both innate and adaptive immunity compared with ibrutinib [20,62,63]. Of note, ibrutinib, unlike other more selective BTKis (acalabrutinib and zanubrutinib), has been proposed as a new predisposing factor for IFIs and incorporated as a novel host factor for the definition of probable invasive pulmonary mold disease by the European Organization for Research and Treatment of Cancers (EORTC) and the Mycoses Study Group (MSG) [64].

**Figure 6:**
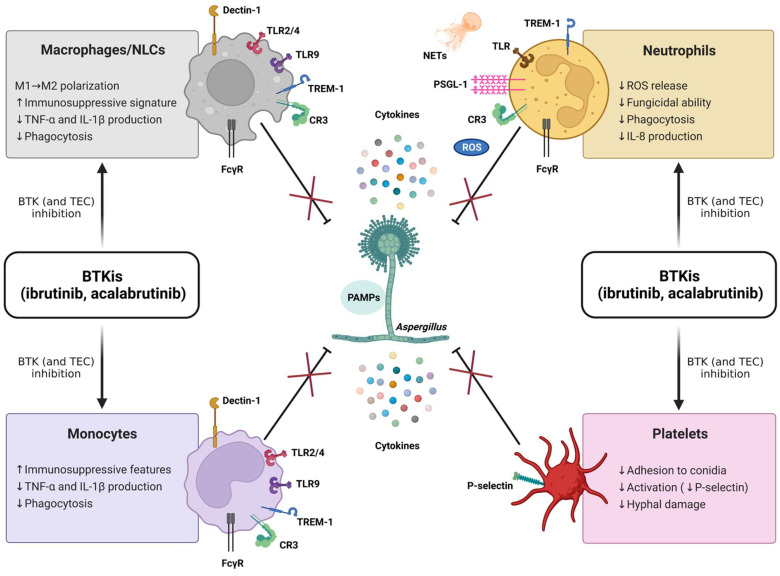
BTK inhibitors (BTKis) suppress (×) antifungal immune responses (⊥) mediated by macrophages, nurse-like cells (NLCs), monocytes, neutrophils, and platelets. The fungal cell wall contains polysaccharides and lipid moieties that elicit an immune response with a strong production of cytokines, comprising tumor necrosis factor α (TNF-α), interleukin 1β (IL-1β), IL-6, and IL-8. The recognition of fungi by innate immune cells relies on the interaction between pathogen-associated molecular patterns (PAMPs, i.e., β-glucans, chitins, and mannans) and different pattern recognition receptors (PRRs). The typical PRRs for *A. fumigatus* encompass β-glucan receptor (Dectin-1), complement receptor 3 (CR3), triggering receptor expressed on myeloid cells-1 (TREM-1), and Toll-like receptors (TLRs). BTK is crucially involved in the transmission of multiple signaling cascades activated by PRRs and, therefore, can be considered a ‘guardian’ of the innate immunity. Ibrutinib and acalabrutinib compromise the ability of neutrophils, monocyte/macrophage populations, and platelets to counteract the fungal growth. Of note, acalabrutinib, unlike ibrutinib, has not yet been associated with an increased risk for invasive fungal infections (IFIs) in treated patients, nor have detrimental effects on antifungal innate immune responses been described in vivo so far. It is conceivable that the presence of functional TEC (not targeted by acalabrutinib) may partially compensate for nonfunctional BTK in non-B cells, as suggested for patients with X-linked agammaglobulinemia (XLA), showing unremarkable rates of IFIs. The combined inhibition of BTK and TEC (both targeted by ibrutinib) is expected to fully suppress BTK/TEC-dependent inflammatory pathways, thus leading to an augmented susceptibility to IFIs. FcγR, Fc-gamma receptor; ROS, radical oxygen species; NETs, neutrophil extracellular traps; PSGL-1, P-selectin glycoprotein ligand 1; ↑, increase; ↓, decrease.

Despite these recent achievements, much remains to be learned about the complex role of BTK within the innate immune system and its potential clinical relevance. In particular, the specific immunopathogenic mechanisms underlying the susceptibility to IFIs in patients treated with BTKis are not fully elucidated, and multiple cell pathways are deemed to be implicated (Figure 6). Relevant to this, by considering the previously described antifungal properties of platelets [65] and the well-known antiplatelet effects of BTKis (classically associated with bleeding complications) [66], it could be argued that an impairment of platelet-mediated antifungal responses may contribute to the emergence of IFIs in CLL patients. Nonetheless, this suggestive hypothesis has never been explored so far, and, more generally, the potential impact of BTKis on innate immune functions of platelets is largely unknown. Intriguingly, Naylor-Adamson et al. have recently reported that platelets from CLL patients can respond to *Staphylococcus aureus* and *Escherichia coli* bacteria in an FcγRIIA-dependent manner and that ibrutinib impairs such responses, highlighting the relevance of evaluating the effects of BTKis on platelet immune functions [67]. In the present work, we confirm the significant role of platelets in the host defense against *A. fumigatus*, and we demonstrate, for the first time, that the exposure to BTKis impairs different antifungal immune functions of platelets. Our original data show that in vitro and in vivo inhibition of BTK, by either ibrutinib or acalabrutinib, suppresses platelet-mediated antifungal activities, with a reduction in platelets’ adhesion to conidia and conidia-mediated P-selectin expression, as well as platelet-induced hyphal damage, thereby supporting the hypothesis that ibrutinib may promote the development of IFIs through a detrimental effect on platelets.

## 5. Conclusions

Moving from bench to bedside, our study reveals specific modifications in antifungal immune responses mediated by platelets induced by ibrutinib and acalabrutinib both in CLL patients and healthy donors, shedding further light on the relationship between treatment with BTKis and IFIs. In particular, BTKi-mediated suppression of platelet immune functions might contribute to mold infections by interfering with the ability of platelets to directly counteract the fungal growth and cooperate with other immune cells.

In perspective, additional studies are required to (i) precisely assess the risk of IFIs occurring in patients treated with ibrutinib and, more importantly, with other BTKis; (ii) disclose whether the multifunctional impairment in innate immunity is strictly caused by the inhibition of BTK outside the BCR (i.e., on unwanted cellular targets expressing BTK) or, more likely, is also mediated by unselective molecular inhibition of other kinases (such as TEC); (iii) fully characterize platelet proteomic and structural modifications occurring in the presence of BTKis; and (iv) test new immunological tools and specific inflammatory biomarkers, including platelet parameters, such as mean platelet volume (MPV) and platelet distribution width (PDW) [68,69], aiming to identify and monitor ‘protective vs. permissive’ immune profiles in patients with or at risk for IFIs.

Finally, by considering that the exposure to BTKis is probably not sufficient to lead to the onset of IFIs, the development of novel score systems (based on all the inherited and acquired host factors, as well as environmental factors) is warranted to refine individual risk stratification, possibly guiding the clinical use of antifungal prophylaxis.

## Data Availability

The data presented in this study are available upon reasonable request.
